# Risk factors and patterns of household clusters of respiratory viruses in rural Nepal

**DOI:** 10.1017/S0950268819001754

**Published:** 2019-10-14

**Authors:** E. M. Scott, A. Magaret, J. Kuypers, J. M. Tielsch, J. Katz, S. K. Khatry, L. Stewart, L. Shrestha, S. C. LeClerq, J. A. Englund, H. Y. Chu

**Affiliations:** 1 School of Medicine, University of Washington, Seattle, WA, USA; 2 Department of Global Health, Milken Institute School of Public Health, George Washington University, Washington, DC, USA; 3 Department of International Health, Johns Hopkins Bloomberg School of Public Health, Baltimore, MD, USA; 4 Nepal Nutrition Intervention Project – Sarlahi, Kathmandu, Nepal; 5 Department of Paediatrics and Child Health, Institute of Medicine, Tribhuvan University, Kathmandu, Nepal; 6 Seattle Children's Hospital and Research Institute, University of Washington, Seattle, WA, USA

**Keywords:** Household transmission, molecular epidemiology, respiratory syncytial virus, respiratory viruses, rhinovirus

## Abstract

Viral pneumonia is an important cause of death and morbidity among infants worldwide. Transmission of non-influenza respiratory viruses in households can inform preventative interventions and has not been well-characterised in South Asia. From April 2011 to April 2012, household members of pregnant women enrolled in a randomised trial of influenza vaccine in rural Nepal were surveyed weekly for respiratory illness until 180 days after birth. Nasal swabs were tested by polymerase chain reaction for respiratory viruses in symptomatic individuals. A transmission event was defined as a secondary case of the same virus within 14 days of initial infection within a household. From 555 households, 825 initial viral illness episodes occurred, resulting in 79 transmission events. The overall incidence of transmission was 1.14 events per 100 person-weeks. Risk of transmission incidence was associated with an index case age 1–4 years (incidence rate ratio (IRR) 2.35; 95% confidence interval (CI) 1.40–3.96), coinfection as initial infection (IRR 1.94; 95% CI 1.05–3.61) and no electricity in household (IRR 2.70; 95% CI 1.41–5.00). Preventive interventions targeting preschool-age children in households in resource-limited settings may decrease the risk of transmission to vulnerable household members, such as young infants.

## Introduction

Acute lower respiratory infection (ALRI) is the primary cause of child morbidity and mortality worldwide with the vast majority of childhood deaths related to ALRI occurring in resource-limited settings [1]. Respiratory viruses are increasingly recognised as a cause of severe ALRI in young children [2]. In many global regions where access to healthcare is limited, especially in rural areas, the true community-based burden of respiratory virus-associated ALRI remains poorly characterised [3–5]. In these settings, household surveillance studies can provide a more comprehensive evaluation of viral incidence, transmission and molecular epidemiology patterns in the community [4–8].

Household surveillance can provide valuable information regarding the transmission networks within households. Such knowledge may guide the development and implementation of preventative interventions to protect vulnerable groups from ALRI. For example, infants are at highest risk for severe ALRI from respiratory syncytial virus (RSV) [9]. Major challenges to developing a safe and effective RSV vaccine in young infants have resulted in the development of alternative strategies including maternal RSV vaccination and delayed vaccine administration until >6 months of age [10]. Targeting older groups for vaccination may protect vulnerable populations by interfering in transmission chains to young infants, the elderly and other high-risk groups.

Studies in rural Kenya have identified school-age children as the primary introducers of RSV into households where an infant subsequently became infected [6]. These results were in agreement with a US study from the 1960s reporting older siblings aged 2–16 years as most likely to introduce RSV disease into families [11]. In contrast, modelling suggests that young children <5 years are more likely to transmit RSV and are the most efficient population to vaccinate in order to prevent disease in other groups [12]. Few studies have analysed the transmission of other non-influenza respiratory viruses, such as human metapneumovirus (MPV) and human rhinovirus (HRV), and no studies have examined the household transmission dynamics of respiratory viruses in rural South Asia, a region characterised by high rates of preterm birth and infant mortality [8, 13–15].

The aims of this analysis were to characterise the transmission of nine non-influenza respiratory viruses within households in rural Nepal and to determine household characteristics associated with the transmission of respiratory viruses. We hypothesise that the presence of school-age children in a household will be associated with an increased risk of transmission.

## Methods

### Study population

This prospective household surveillance study was nested within a randomised controlled trial designed to determine the effectiveness of influenza vaccine during pregnancy [16, 17]. The study site is in the low-lying region of rural southern Nepal called the ‘terai’, with inhabitants broadly representative of the population of India, Bangladesh and Nepal [18]. All women of childbearing age in a part of one district were surveyed for pregnancy. Pregnant women were enrolled in the primary trial as early as possible in pregnancy, generally during the second trimester, and followed until 6 months postpartum. At the time of randomisation, every third study mother and their families were selected to participate in a household surveillance substudy. As randomisation occurred at the time of vaccination, not initial enrolment, randomisation occurred after enrolment and the start of surveillance for some mothers. Surveillance for the first participant enrolled in the household substudy began on 14 April 2011 and we included the households of substudy mothers enrolled prior to 1 May 2012. Surveillance of the household ended 180 days after birth. In this area, many households consist of multiple families living in a single compound; households were defined as a group sharing a single cookstove. Socio-demographic data were collected upon enrolment at the individual and household levels. Birth assessments of study infants were performed shortly following birth. Infants weighed within 72 h of the birth were considered to be low birthweight if the infant weighed <2.5 kg.

### Sample collection and virological methods

Trained field staff visited the home weekly and used a standardised form to inquire about respiratory symptoms and signs in mothers, infants and other household members for each day in the previous week. A mid-nasal turbinate swab was collected from mothers and other adult household members aged ⩾15 years with self-reported fever, plus one or more of the following symptoms within the previous 7 days: cough, sore throat, runny nose, nasal congestion or myalgias. Swabs were collected from all children <15 years with at least one of the following symptoms: subjective fever, cough, draining ear, wheezing or difficulty breathing, in the previous 7 days. Illness episodes were defined as symptoms that met described criteria and were separated by at least seven symptom-free days. Only individuals with ⩾7 days of symptom diary recorded, with or without illness, were included in the analyses. Households that did not have ⩾3 individuals with surveillance were excluded from the analysis as two-person households consisted of mother–infant pairs without surveillance of household members. Respiratory swabs were collected, aliquoted and transported from the Nepal field site to the University of Washington in Seattle, WA in a temperature-stable buffer (PrimeStore; Longhorn Diagnostics, San Antonio, USA).

Samples were tested by a real-time reverse transcriptase polymerase chain reaction (PCR) for 12 respiratory viruses, including RSV, MPV, influenza viruses A and B, parainfluenza virus 1–4 (PIV 1–4), adenovirus (AdV), human coronavirus (CoV), HRV and bocavirus [19–21]. Influenza transmission in household was the primary aim of the trial substudy and is being analysed separately. Bocavirus was not included because of its prolonged shedding patterns. Sequencing was performed for HRV- and RSV-positive samples from household illness clusters utilizing samples with PCR cycle threshold values <33 and 30 for HRV and RSV, respectively, based on previous difficulty sequencing low viral load samples [22, 23]. Briefly, nucleic acid was extracted, and cDNA was synthesised. A hemi-nested PCR protocol was used targeting the 5′ untranslated region and the second hypervariable region of the attachment (G) glycoprotein coding region for HRV and RSV, respectively [22, 23]. Sequences were aligned using MAFFT v7.309, and maximum likelihood phylogenetic trees using the HKY85 model with 100 bootstrap replicates were inferred using PhyML 3.1 within Geneious [24, 25]. Sequences were considered to be the same virus type with ⩾98% identity. Sequences >200 base pairs (bp) were submitted to GenBank under accession numbers MH266546 to MH266612.

### Statistical analysis

For each examination of viral transmission, initial viral infections were those preceded by a 14-day period without infections of that virus type in a household. Index or transmitting cases were established by identifying the individual(s) with the earliest reported respiratory symptoms prior to a virus-positive swab. We computed the incidence of secondary household illness cases following each initial viral infection. We defined transmission events as observed infections of the same virus in another member of the same household within the subsequent 14 days. The 14-day risk period began on the first day of criteria respiratory symptoms associated with virus-positive specimen collection in the week preceding the virus-positive swab in the index case. Each initial infection and its corresponding risk period comprise a single data point in the regressions and include subsequent infections and time at risk from all household members reporting symptoms over that time period. To assess the risk factors for the incidence of secondary cases of these initial illnesses within households, we used generalised estimating equations, accounting for the potential similarity among repeated initial infections within households. The outcome was the number of secondary cases, with a fixed offset of the log time at risk; a log link was used to estimate the incidence rate ratios (IRRs). Multivariable regression was performed by first including all measures significant in the univariable analysis at *P* < 0.1 and then performing backward elimination to select a final model. Measures were retained in final multivariable regression if significant at *P* < 0.05 or if had a substantial impact (⩾10% shift in estimate) on other significant covariates. A single index case type was included in the multivariable model as the index case was coded such that one index case type was compared to all other index cases.

Potential risk factors for transmission included maternal and household characteristics. Some households included more than one enrolled mother–infant pair. Among households with more than two mothers, household characteristics were compared with a sensitivity analysis using one mother's descriptors *vs.* the others. Data were analysed using SAS/STAT 9.4 (SAS Institute Inc.) and Stata 15 (STATA Corp) statistical software.

### Human subjects

Institutional review board approval for the randomised controlled trial was given by the Johns Hopkins University Bloomberg School of Public Health, Cincinnati Children's Hospital, the Institute of Medicine at Tribhuvan University and the Nepal Health Research Council, with deferral from Seattle Children's Hospital. Approval for this analysis was received from the University of Washington institutional review board. The primary trial was registered under ClinicalTrials.gov NCT01034254.

## Results

### Population characteristics

**Fig. 1.**
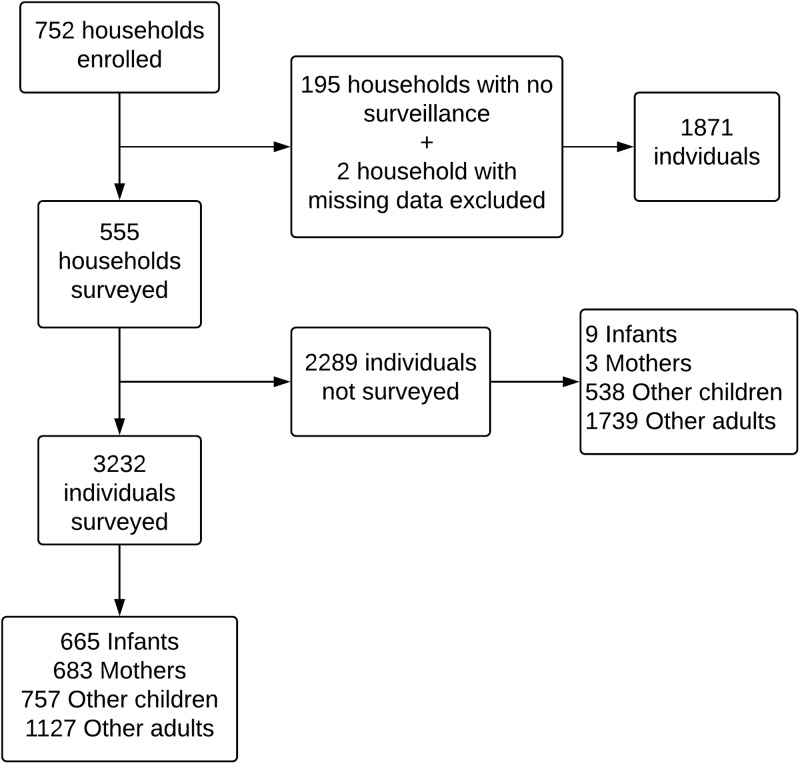
Summary of household and individual enrolment, surveillance and inclusion in the analysis of respiratory virus transmission in households in Sarlahi, Nepal.

A total of 752 households were enrolled with a median household size of 9 (range 2–31). Five-hundred and fifty-five households contributed symptom reporting from at least three persons. Within the 555 surveyed households, 3232 out of 5521 (59%) initially enrolled household members were surveyed for weekly respiratory illness. These 3232 individuals included in the transmission analysis consisted of 683 mothers, 665 infants, 1127 other adults ⩾15 years and 757 other children <15 years (Fig. 1). Characteristics of all households and individual characteristics of surveyed individuals are summarised in Table 1. Within included households, 99% of study mothers and infants were surveyed, whereas only 39% of other adult household members and 58% of other children were surveyed. The proportion of other adults with surveillance was 32% *vs.* 53% among individuals <40 *vs.* ⩾40 years, and 37% *vs.* 43% in males compared to females. Forty-nine per cent of other children aged 5–14 years were surveyed compared to 73% of other children aged <5 years. Of children aged 5–14 years attending school, 65% were surveyed compared to 49% of non-school attending children.

**Table 1.** Demographic and clinical characteristics of households in Sarlahi district, Nepal

Household characteristics	Households with surveillance (*n* = 555)^a^	Households without surveillance (*n* = 195)^a^
Household crowding >4 people per room	207 (38)	63 (33)
Electricity in household	501 (91)	172 (89)
Latrine in household	272 (49)	100 (52)
Smoker in household	261 (47)	88 (46)
Mother with ⩾1 year formal education	306 (57)	102 (56)
Caste		
Brahmin	56 (10)	20 (10)
Vaishya	310 (56)	96 (50)
Other	187 (34)	77 (40)
Ethnicity		
Pahadi	325 (59)	110 (57)
Madhesi	228 (41)	83 (43)
Individual characteristics	Surveyed household members (*n* = 3232)^a^	
Study mothers	683 (21)	
Age at enrolment, years	22 (14, 41)	
Years of education	5 (0, 16)	
Study infants	665 (21)	
Male, sex	303 (55)	
Low birthweight^b^	108/439 (25)	
Prematurity^b^	72 (13)	
Small for gestational age^b^	170/441 (39)	
Other children <5 years	382 (12)	
Other children aged 5–14 years	375 (12)	
Children 5–14 years attending school	294 (78)	
Other adults ⩾15 years	1127 (35)	
Male, sex	642 (55)	
Age at enrolment, years	37 (16, 97)	

a *N* (%) or median (range).

b Prematurity was defined as gestational age <37 weeks. Low birthweight was defined as <2500 g in infants whose weight was measured within 72 h of birth. Small for gestational age was based on intergrowth 21 criteria.

### Household-level transmission

**Fig. 2.**
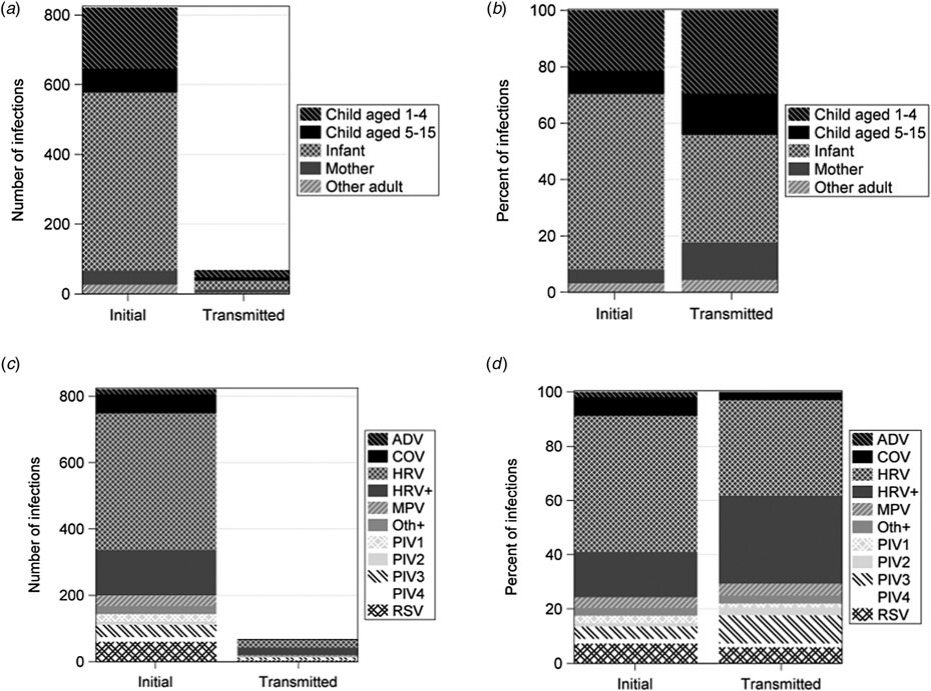
All initial illness episodes *vs.* initial episodes resulting in any transmission by proportions and counts. (Upper) Illness episodes compared by household member type of index case in counts (a) and proportion of episodes (b). (Lower) Illness episodes compared by virus of index case in counts (a) and proportion of episodes (b). HRV + represents coinfection of HRV and 1 + other virus. Oth + represents coinfection not involving HRV.

**Fig. 3.**
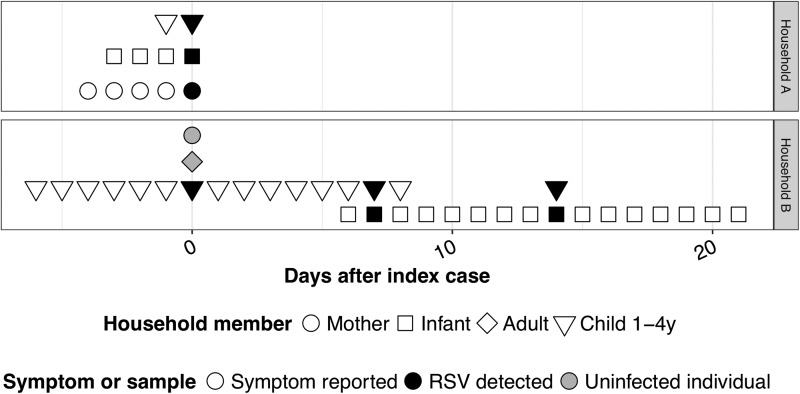
Examples of symptoms and RSV-positive specimen collection in two examples of RSV infection clusters in two households (a and b). Each row represents an individual, each unfilled symbol represents 1 day of symptoms, black filled symbols represent positive specimen collection and varying symbols represent household member type. Index cases are those whose symptoms first appear before the initial RSV-positive specimen.

A total of 825 virus-positive initial illness episodes occurred within 362 households with a median of one (range 0–10) illness episode per household. In the 14 days following initial household illness, 110 subsequent illness episodes occurred, 88 (80%) of which were screened by PCR and 22 (20%) illnesses that did not have a swab collected despite meeting symptom criteria. Eight per cent of illness episodes resulted in a PCR-confirmed secondary case within the household with a total of 79 transmission events in 68 household illness episodes. Household illness clusters occurred in 58 households as some households experienced multiple illness clusters. The incidence of a PCR-confirmed transmission event of any virus occurring in the 14 days following initial infection was 1.14 transmissions per 100 person-weeks. The index or transmitting case was most frequently a 1–4 years old child (*n* = 33; 41.8%) – referred to subsequently as preschool children – followed by infants (*n* = 28; 35.4%). Twenty (11.4%) illness episodes in preschool children and 26 (5.1%) in infants resulted in a secondary illness case. In 36 any-virus transmission events, infants were a secondary case, representing 45.6% of all transmission events and 7.0% of all infant illness episodes (Table 2; Fig. 2). Preschool age children were the second most common secondary case in 30 (38%) any-virus transmission events. In RSV transmission events, infants were identified as the index case most commonly in eight (61.5%) events, followed by preschool children in six (46.2). An infant was the secondary case in nine (69.2%) RSV transmission events (Table 2; Fig. 3). HRV coinfection with another respiratory virus had more frequent transmissions (16.1% of HRV coinfections resulted in transmission) compared to monoinfection of HRV (5.8%), coronavirus (3.5%) and RSV (6.7%) (Fig. 2, see Table 3 for statistical testing).

**Table 2.** Household transmission episodes of nine respiratory viruses in households in Sarlahi district, Nepal

	RSV	MPV	HRV	CoV	PIV-1	PIV-2	PIV-3	PIV-4	AdV	Total^a^
1st episodes (households)	106 (100)	81 (80)	560 (307)	107 (89)	30 (28)	16 (14)	68 (62)	26 (26)	36 (33)	825 (362)
Episodes with transmission (households)	10 (10)	6 (6)	43 (30)	4 (4)	1 (1)	4 (4)	13 (11)	2 (2)	0	68 (58)
Transmission events	13	8	43	4	1	4	13	2	0	79
Transmission incidence per 100 *p*-weeks	1.53	1.09	0.93	0.43	0.38	2.71	2.19	0.96	0	1.14
Median (IQR) Household members, enrolled	9 (6, 13)	10 (7, 13)	9 (6, 12)	9 (6, 13)	10 (6, 11)	10 (6, 11)	9 (8, 12)	9 (6, 11)	10 (6, 13)	9 (6, 12)
Median (IQR) Household members, follow-up within 14 days of index infection	4 (3, 6)	4 (3, 6)	6 (3, 9)	5 (3, 7)	4 (4, 7)	6 (4, 6)	5 (3, 6)	4 (3, 6)	4 (3, 5)	7 (4, 13)
Median serial index, days (range)^b^	3 (0, 21)	3 (0, 13)	2 (0, 14)	4 (3, 8)	8	7 (3, 10)	4 (0, 14)	1 (1, 1)	–	3 (0, 14)
Days to transmission > 0	7 (53.9)	7 (87.5)	38 (88.4)	4 (100.0)	1 (100.0)	4 (100.0)	10 (76.9)	2 (100.0)	–	68 (86.1)
Index cases, *n* (%)										
Infant	8 (61.5)	2 (25.0)	12 (27.9)	1 (25.0)	0 (0.0)	0 (0.0)	8 (61.5)	1 (50.0)	–	28 (35.4)
Mother	2 (15.4)	2 (25.0)	7 (16.3)	0 (0.0)	0 (0.0)	0 (0.0)	1 (7.7)	0 (0.0)	–	11 (13.9)
Other children	8	5	26	2	1	4	5	1	–	45
Child <5 years	6 (46.2)	5 (62.5)	17 (39.5)	2 (50.0)	1 (100.0)	2 (50.0)	5 (38.5)	1 (50.0)	–	33 (41.8)
Child 5–14 years	2 (15.4)	0 (0.0)	10 (23.3)	0 (0.0)	0 (0.0)	2 (50.0)	1 (7.7)	0 (0.0)	–	14 (17.8)
Other adult	1 (7.7)	0 (0.0)	1 (2.3)	1 (25.0)	0 (0.0)	0 (0.0)	1 (7.7)	0 (0.0)	–	3 (3.8)
Secondary cases, *n* (%)										
Infant	9 (69.2)	3 (37.5)	20 (46.5)	3 (75.0)	0 (0.0)	3 (75.0)	4 (30.8)	1 (50.0)	–	36 (45.6)
Mother	0 (0.0)	1 (12.5)	5 (11.6)	1 (25.0)	1 (100.0)	0 (0.0)	2 (15.4)	0 (0.0)	–	8 (10.1)
Other children	10	4	20	0	0	1	6	1	–	39
Child <5 years	7 (53.9)	4 (50.0)	14 (32.6)	0 (0.0)	0 (0.0)	0 (0.0)	6 (46.2)	1 (50.0)	–	30 (38.0)
Child 5–14 years	3 (23.1)	1 (12.5)	7 (16.3)	0 (0.0)	0 (0.0)	1 (25.0)	1 (7.7)	0 (0.0)	–	11 (13.9)
Other adult	0 (0.0)	0 (0.0)	1 (2.3)	0 (0.0)	0 (0.0)	0 (0.0)	3 (23.1)	0 (0.0)	–	4 (5.1)

a Total for initial episodes, transmission episodes and transmission events are less than the sum of columns as infections with coinfections were counted a single initial infection in total. Similarly, the total for index cases may be less than the sum of columns as coinfection transmission or transmission to multiple household members was only counted once in total.

b Median serial index was defined as the median number of days between symptom onset of index and secondary case.

**Table 3.** Univariable and multivariable regression using general estimating equations to associate household characteristics with the incidence of household transmission of respiratory viruses in Sarlahi district, Nepal.

	Factor prevalence	Transmission incidence per 100 *p*-weeks		Univariate analysis		Multivariate analysis	
Household demographic characteristics		Factor absent	Factor present	IRR (95% CI)	*P*-value	IRR (95% CI)	*P*-value
Electricity in house	92% (3055/3337)	**2.53**	**1.22**	**0.42 (0.22–0.78)**	**0.006**	**0.37 (0.20–0.71)**	**0.0027**
Latrine in house	38% (1247/3315)	1.53	0.97	0.80 (0.41–1.56)	0.52		
Mother with any formal education	45% (1389/3091)	1.51	1.16	0.82 (0.46–1.48)	0.51		
Open biofuel cookstove use	91% (3052/3346)	1.41	1.36	1.06 (0.44–2.55)	0.89		
Household crowding above 4 people per room	51% (1690/3335)	1.23	1.49	1.02 (0.57–1.82)	0.95		
Any member of household smokes	54% (1816/3344)	1.55	1.18	0.90 (0.50–1.61)	0.72		
Infant in household							
Male infant	55% (1855/3346)	1.11	1.59	1.48 (0.84–2.60)	0.18		
Low birthweight infant	31% (853/2773)	1.01	2.24	**2.06 (1.13–3.76)**	**0.019**		
Prematurity	12% (402/3346)	1.24	2.22	1.80 (0.94–3.45)	0.078		
Small for gestational age	44% (1211/2773)	1.21	1.54	1.29 (0.70–2.37)	0.42		
Index infection was infant	59% (1982/3353)	**2.53**	**0.74**	**0.35 (0.20–0.62)**	**0.0003**		
Index infection was child aged 1–4 years	24% (789/3353)	**0.98**	**2.64**	**2.33 (1.50–3.62)**	**0.0002**	**2.35 (1.40–3.96)**	**0.0012**
2 + children aged 1–4 years in household	54% (1795/3344)	**0.88**	**1.90**	**1.90 (1.05–3.46)**	**0.035**		
2 + children attending school	47% (1582/3344)	1.24	1.50	0.96 (0.54–1.73)	0.90		
Initial infection was coinfection	20% (161/825)	**1.23**	**1.91**	**2.15 (1.26–3.66)**	**0.005**	**1.94 (1.05–3.61)**	**0.035**

The regression examines risk factors at the incident infection level, thus there are 825 rows in the regression analysis dataset. Note that 3353 is the number of persons at risk of transmission from the original 825 illness episodes.

Significant results are shown in bold, *α* 0.05.

### Risk factors for transmission

We evaluated risk factors for transmission following initial respiratory viral illness (Table 3). In bivariate analyses, an infant index case was a protective factor associated with decreased transmission incidence (0.7 *vs.* 2.5 events per 100 person-weeks; IRR 0.35; 95% confidence interval (CI) 0.20–0.62), as was electricity in the household (1.2 *vs.* 2.5; IRR 0.42; 95% CI 0.22–0.78). Increased incidence of transmission was associated with a preschool-age child index case (2.6 *vs.* 1.0; IRR 2.33; 95% CI 1.50–3.62), ⩾2 preschool children in the household (1.9 *vs.* 0.9; IRR 1.90; 95% CI 1.05–3.46), a low birthweight infant in the household (2.2 *vs.* 1.0; IRR 2.06; 95% CI 1.13–3.76) and viral coinfection as the transmitting infection (1.9 *vs.* 1.2; IRR 2.15; 95% CI 1.26–3.66). Following backward elimination, preschool-age child as index case, electricity in the household and initial infection with coinfection were retained in the multivariable analysis. In the multivariable model, preschool child index case (IRR 2.35; 95% CI 1.40–3.96), electricity in household (IRR 0.37; 95% CI 0.20–0.71) and initial coinfection (IRR 1.94; 95% CI 1.05–3.61) were significantly associated with transmission.

### Sequencing

**Fig. 4.**
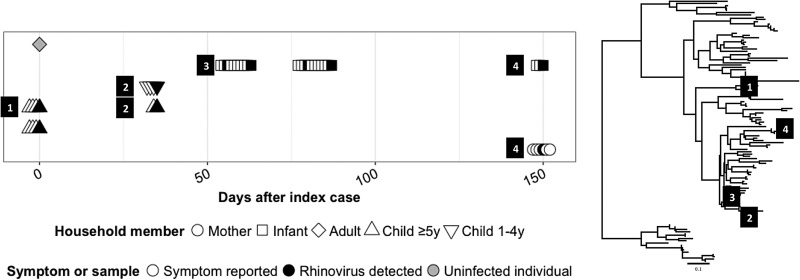
Multiple HRV infection clusters within a single household. (Left) Timeline of symptoms (empty symbols) and HRV-positive specimen collection (black symbol) with each row representing one household member. (Right) HRV phylogenetic tree. Numbered squares on the phylogenetic tree represent an HRV genotype and correspond to numbered squares to left of the HRV specimen on the illness timeline. HRV specimens without a numbered square were not sequenced.

Sequencing of RSV and HRV samples involved in transmission episodes was used to determine if individuals were infected with the same viral strain. Eight of 15 RSV samples (53.3%) were successfully sequenced with a median sequence length (range) of 418 base pairs (208–470) (Supplementary Fig. S1). Sequences matched in two of three fully evaluated transmission events (22.2% of all RSV transmission events). Sequencing was attempted for 33 (76.7%) HRV transmission events and was successful in 40 of 78 (51.3%) specimens with a median sequence length (IQR) of 204 bp (166–239) (Supplementary Fig. S2). Of nine fully evaluated HRV transmission events, household sequences matched in six (18.1% of all HRV transmission events). In three events (9.1% of all transmission events), the individuals were infected with different HRV genotypes (Fig. 4). All other episodes had insufficient data to confirm the transmission of specific viral genotypes.

## Sensitivity analyses

Of 362 households contributing to the regression analysis of any virus transmission, 52 households included more than one mother–infant pair. Within households with multiple mothers, there was 100% agreement in reporting of electricity within the home, 89% agreement in indoor cookstove use and 85% agreement of latrine within the home. The multivariable regression model was performed using the alternative mother's household information with similar results (Supplementary Table S1). A sensitivity analysis was also performed using a definition of transmission as a secondary case of the same virus within 28 days of initial infection within the household. Using this definition, preschool child index case, coinfection as initial infection and a low birthweight infant in the household were associated with an increased incidence of household transmission (Supplementary Tables S2 and S3).

## Discussion

In a prospective longitudinal study utilizing intensive weekly home-based active surveillance to evaluate the household transmission of nine respiratory viruses in rural South Asia, initial infection in young children was associated with the greatest risk of symptomatic respiratory virus household transmission with spread to infants occurring in 45% of transmission events. Southern Nepal is a region where household crowding is common and there are high rates of infants born prematurely or low birthweight [26, 27]. Our data demonstrate a significant burden of symptomatic respiratory viral illness in households; based on a multivariable model, young children and socio-cultural factors, such as socio-economic status, may predispose to the transmission of viruses in this region.

In over 40% of transmission events of all viruses, preschool children (aged 1–4 years) served as an index case. A higher proportion of initial infection among this group resulted in secondary cases compared to other age groups, including school-age children and mothers, a finding confirmed in our multivariable model of transmission incidence. In RSV transmission, no index cases were older children and 15% of index cases were mothers. In contrast to our findings, a study of RSV transmission in the USA during the 1960s found that older siblings between 2 and 16 years most frequently introduced RSV into a household [11]. Similarly, a Kenyan household study examined sequencing-confirmed RSV transmission in 44 households with infants and identified school-age children as the most common index case resulting in infant infection [6].

Our finding that preschool-age children, rather than school-age children, are most likely to transmit non-influenza respiratory viruses is likely due to differences in study sample and design, as well as transmission patterns. Households in our study experienced fewer respiratory viral illness episodes than reported in other household studies that included asymptomatic viral detections [5, 7, 15]. In a study of respiratory virus-positive influenza-like illness in households in Vietnam, households experienced 1.6 illness episodes over a 1-year period (including influenza and bocavirus), whereas we found a mean of 1.4 illness episodes per household [8]. Our surveillance sample includes a higher proportion of young children aged 0–4 years, including study infants, relative to the overall proportion in the Sarlahi district of Nepal (32.4% *vs.* 11.3%) and a lower proportion of children aged 5–14 years (11.6% *vs.* 27.9%) [18]. It is possible that the true transmitting cases were absent during the weekly household visit or asymptomatic according to our criteria, though this is less likely for younger children who have median viral shedding duration of longer than 1 week [31]. We likely did not capture the full contribution of older children to transmission compared to the Kenyan cohort. Over half of RSV infections in children 5–15 years in that study were asymptomatic, with a smaller proportion of asymptomatic infections in infants under 1 year and children 1–4 years at 9% and 17%, respectively [28]. However, they also reported viral shedding in symptomatic RSV infections was 14 log_10_ RNA copies greater than in asymptomatic RSV cases suggesting that symptomatic episodes are more likely to transmit virus [29]. Last, we collected weekly specimens and our findings may be biased if non-infant younger children had longer shedding duration compared to older children. However, this has not been demonstrated in studies of RSV and HRV shedding duration [28, 30].

Our large cohort allowed us to use a multivariable analysis to identify the risk factors and protective characteristics associated with the incidence of transmission. While both infants and preschool children were frequently identified as the index case in a transmission event and can shed virus for prolonged periods, a preschool child index case was associated with a twofold increased risk of transmission and an infant index case was associated with a decreased risk of transmission [11, 31, 32]. Whereas infants are more likely to transmit RSV via direct contact as compared with fomites, young children may transmit infection efficiently through both methods due to differences in mobility and behaviour [33]. Coinfection as the initial infection was associated with an increased risk of transmission, including in our multivariable model. Coinfections most commonly involved HRV and a greater proportion of coinfections resulted in a secondary case compared to monoinfection of most viruses. Viral coinfection with RSV infection has been demonstrated to increase RSV viral load and shedding duration. However, this has not been consistently seen, including a study analysing seven respiratory viruses [29 31, 34]. Finally, electricity in the household, a proxy for socio-economic status and housing conditions, was negatively associated with transmission. Although an association between indoor air pollution and RSV infection has been reported in resource-limited regions, smoking and biofuel cookstove use were not associated with the risk for transmission in our model [4]. However, we had limited power to detect this association due to the use of indoor biofuel cookstoves in over 90% of households in our model and exposures were self-reported without actual measures of indoor air pollution.

A study in Peru demonstrated that age, occupation and household size can influence contact network size and pattern [35]. Our findings, from a population consisting of crowded households, lower levels of maternal education and fewer children attending school compared to other household transmission studies, suggest that differences in socio-demographic, cultural and environmental contexts influence household transmission risk factors, including the source of household introduction. As we actively surveyed all women of childbearing age for pregnancy, our cohort is generalisable to households with young infants in Southern Nepal, a region representative of rural South Asia [17]. In the Sarlahi district during the study period, an estimated 25% of the population were below the poverty line and approximately 30% of infants were born low birthweight and 20% preterm [26, 27]. Households in our study were crowded with over one-third containing >4 people per room and multiple family units frequently living in a single structure. The average population is young; the median age in the Sarlahi district was 20 years [18]. Eighty-four per cent of households used indoor biofuel cookstoves and half contained latrines. Young demographics, crowded housing conditions and socio-economic factors may influence the patterns of respiratory virus transmission we observed. Transmission may also be affected by the social structures of households and the pattern of migrant labour in this region, where many young men are working outside Nepal, mostly in the Middle East. Twenty per cent of the total Nepalese population were reported as absent from the home, including one-third of 15–29 years old and one-fourth of 30–34 years old, 90% of whom were male [36].

This socio-demographic, environmental and cultural context should be considered when implementing preventative strategies for the control of respiratory viral illness, such as vaccines, antivirals, hygienic measures and physical barriers. For example, there are multiple RSV vaccines targeting diverse populations from infants and children to pregnant women and other adults in various stages of clinical trials [10]. Because the immune systems of neonates generally do not respond well to primary vaccination, immunizing mothers and other household members has been proposed as a method to protect vulnerable young infants from RSV [37]. While a model of Kenya transmission data supports immunizing school-age children to diminish transmission of the virus to infants, our study suggests that in rural South Asia, preschool-age children are more likely to transmit respiratory viruses to other household members [38]. This suggests that a ‘one-size fits all’ approach to RSV vaccine implementation, or other respiratory viral transmission prevention measures, may not be effective as transmission dynamics may differ across global settings.

Our study has several limitations. Asymptomatic infections were not captured, affecting our ability to fully characterise the transmission chain. We expect that asymptomatic transmission may have impacted our ability to characterise HRV spread, particularly in transmission involving older children and adults, our ability to associate age of index case with transmission risk [30, 39]. Moreover, we likely only captured a minority of adult illness as adults required subjective fever for specimen collection and fever occurs infrequently in adult RSV, MPV and HRV illness [7 40]. While we underestimated transmission, specifically spread involving individuals ⩾5 years, due to our symptom criteria, a previous study of RSV transmission demonstrated that the odds of transmission in symptomatic infection is five times that of asymptomatic infections [28]. This suggests that we likely captured index infections. We anticipate that some illness with shedding <7 days may have been missed due to our weekly surveillance. We expect that this represented a small minority of illness episodes as the estimated shedding duration of HRV and RSV in adults is 10 and 9 days, respectively [30, 31]. Shedding for 1–2 weeks is common with paediatric respiratory infections [31]. Moreover, while we performed sequencing on RSV and HRV samples involved in transmission chains, we were not able to phylogenetically verify transmission in the majority of episodes due to high cycle threshold values, and could not use sequencing data to define transmission. Our sequencing results revealed a degree of misclassification with some HRV and RSV transmission events representing illness clusters with multiple virus types circulating in the household simultaneously. This was especially true for HRV, a finding in agreement with previous studies of HRV transmission, including in household and daycare settings [30, 39 41]. Additionally, some households originally selected for the household substudy were not surveyed as intended. Individuals within selected households who were not surveyed were primarily adults. Among adults, males and those <40 years old were surveyed less frequently, a group not considered high risk for household transmission of respiratory viruses in previous studies. A significant proportion of the Sarlahi population, especially men, are reported as absent from home, supporting the possibility that some household members were absent from the community during the study, although these data were not captured [36]. We also surveyed a higher proportion of preschool children compared to school-aged children. The differential exclusion of these subsets may have affected our results, including identification of index cases, especially if these persons were periodically present in the household. Lastly, we did not collect data on the social mixing patterns of individuals in these households which would have provided valuable information regarding possible causal explanations for our findings.

These results provide data that may help to optimise the implementation of preventative strategies with the aim of protecting vulnerable infants. South Asia is an area of the world with a high incidence of low birthweight infants, household crowding and malnutrition, all risk factors for severe childhood ALRI. Our study of non-influenza respiratory virus transmission within households in rural Nepal highlights the importance of targeting preschool-age children to prevent the spread of respiratory viral illness. Understanding the household transmission of respiratory viruses in rural resource-limited populations will help evaluate infection prevention strategies, such as immunisation of mothers and other household members in protecting infants, who are most vulnerable to respiratory viral infection.

## Supplementary material

For supplementary material accompanying this paper visit https://doi.org/10.1017/S0950268819001754. click here to view supplementary material
