# Supplementary figures and images for: Risk factors and patterns of household clusters of respiratory viruses in rural Nepal
Source: Epidemiol Infect. 2019 Oct 14;147:e288. doi: 10.1017/S0950268819001754 (PMC6805793; doi:10.1017/S0950268819001754)

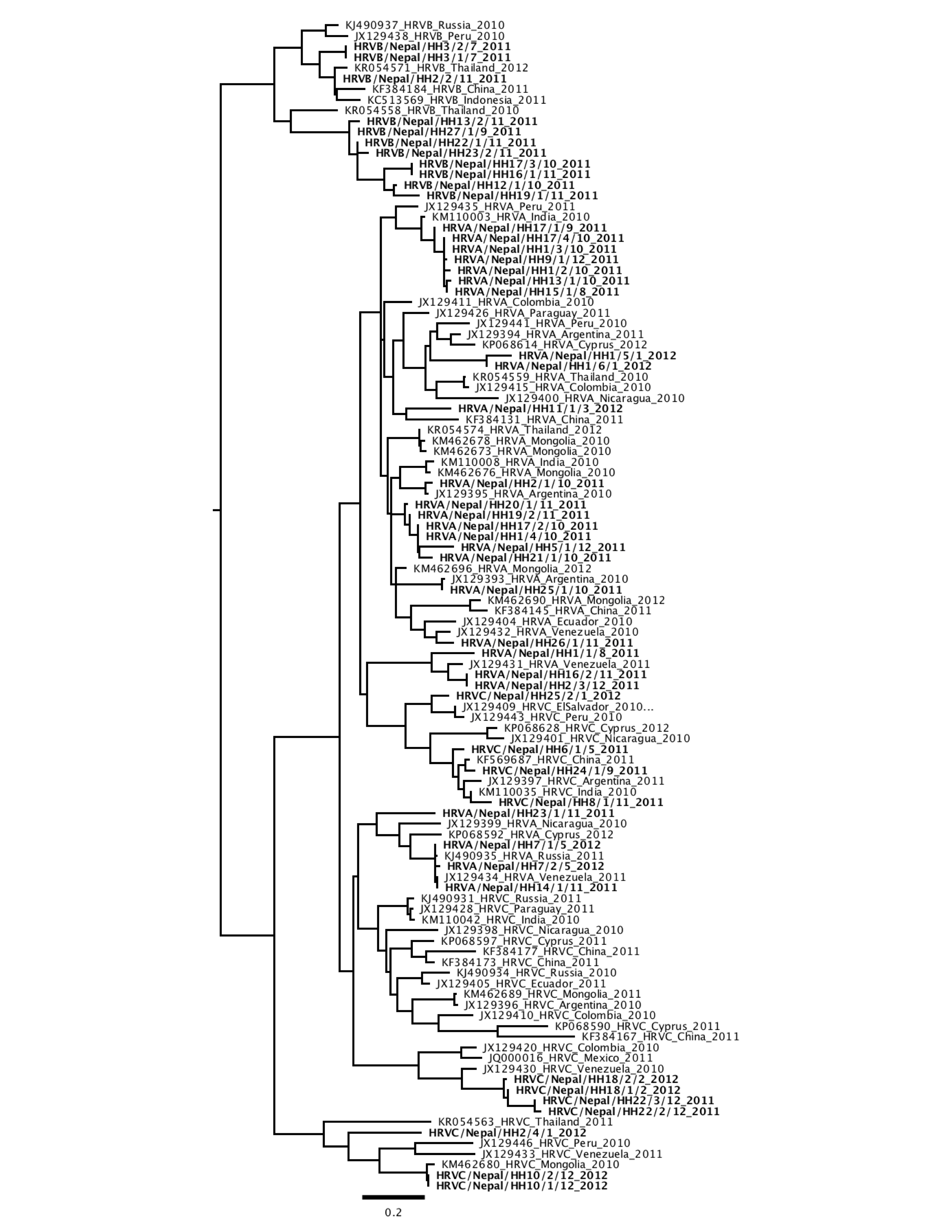

Supplement: Supplementary file 1 [file S0950268819001754sup001.zip › Supplementary Figure 2.tiff]

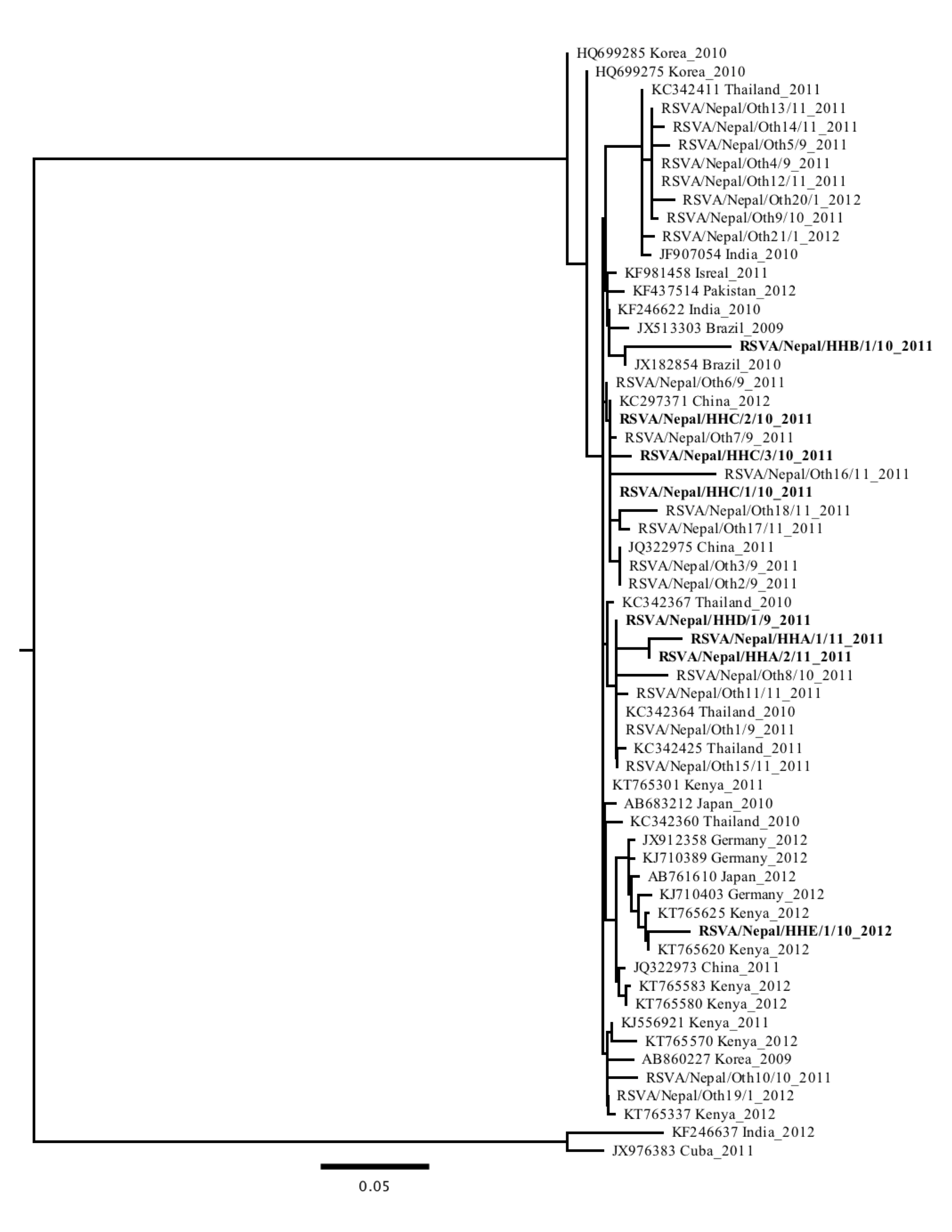

Supplement: Supplementary file 1 [file S0950268819001754sup001.zip › Supplementary Figure 1.png]
